# Health Challenges in Refugee Reception: Dateline Europe 2016

**DOI:** 10.3390/ijerph14121484

**Published:** 2017-11-30

**Authors:** Brad K. Blitz, Alessio d’Angelo, Eleonore Kofman, Nicola Montagna

**Affiliations:** London School of Economics, Middlesex University, The Burroughs, London NW4 4BT, UK; a.dangelo@mdx.ac.uk (A.A.); e.kofman@mdx.ac.uk (E.K.); n.montagna@mdx.ac.uk (N.M.)

**Keywords:** refugee reception, health, medical, psychological services, Italy, Sicily, Greece, European Union

## Abstract

The arrival of more than one million migrants, many of them refugees, has proved a major test for the European Union. Although international relief and monitoring agencies have been critical of makeshift camps in Calais and Eidomeni where infectious disease and overcrowding present major health risks, few have examined the nature of the official reception system and its impact on health delivery. Drawing upon research findings from an Economic and Social Research Council (ESRC) funded project, this article considers the physical and mental health of asylum–seekers in transit and analyses how the closure of borders has engendered health risks for populations in recognised reception centres in Sicily and in Greece. Data gathered by means of a survey administered in Greece (300) and in Sicily (400), and complemented by in-depth interviews with migrants (45) and key informants (50) including representatives of government offices, humanitarian and relief agencies, NGOs and activist organisations, are presented to offer an analysis of the reception systems in the two frontline states. We note that medical provision varies significantly from one centre to another and that centre managers play a critical role in the transmission of vital information. A key finding is that, given such disparity, the criteria used by the UNHCR to grade health services reception do not address the substantive issue that prevent refugees from accessing health services, even when provided on site. Health provision is not as recorded in UNHCR reporting but rather there are critical gaps between provision, awareness, and access for refugees in reception systems in Sicily and in Greece. This article concludes that there is a great need for more information campaigns to direct refugees to essential services.

## 1. Introduction

By the end of 2015, an estimated 1,008,616 people had reached Europe by sea. More than 84% of them were from refugee-producing countries including Syria (49%), Afghanistan (21%) and Iraq (9%). An estimated 17% were women and 25% were children under the age of 18 [1]. In spite of much research on the nature of flows [2,3,4,5,6,7] (and possible incentives to curb irregular migration), there has been little academic work published on conditions in migrant reception centres and even less recorded on the subject of well-being.

International relief and monitoring agencies [8] have been particularly critical of informal camps where infectious disease and overcrowding present major health risks of epidemic proportions [9,10,11]. However, we know much less about attempts to mitigate health risks inside formal reception centres. Given the extensive coverage of the Mediterranean migration “crisis” and the considerable investment made by donor states to the UNHCR, its partners and the governments of principal receiving states, this omission raises several important questions, not least why have scholars neglected this area of investigation? This question takes on added relevance when we consider that not only is there a significant body of related literature but donor policies, especially in the UK, prioritise vulnerable groups, which are taken to include women and children.

Previous research has found that forcibly displaced people face multiple emotional, psychological and medical hardships, as well as nutritional deficiencies [12,13,14,15,16]. Clinical studies of refugees in general reveal that many suffer serious mental health problems as a result of forced displacement, such as depression, schizophrenia, post-traumatic stress disorder, psychosis and suicidal tendencies (see Beiser et al. [12,17,18]). Evidence from past displacement contexts suggests that flaws in the reception system, above all overcrowding, may exacerbate health concerns. As Porter and Haslam record [19], institutional accommodation in post-displacement situations tends to have a strong and negative impact on health outcomes. Further, Porobić has found that, while displacement generates threats to the well-being of refugees, social interventions and collective responses may in fact mitigate the experience of trauma and promote better health [20]. Evaluations of health conditions inside reception centres may therefore inform the delivery of humanitarian assistance and ease integration in the host state.

The increased presence of women and children in Mediterranean flows further makes the case for a deeper investigation into the operation of the reception system and its bearing on health outcomes. Previous studies have indicated lower levels of mental health among female refugees which has been attributed to a host of social factors including low socioeconomic status, un- and underemployment, marginalization, discrimination, language barriers, cultural differences, social stigma, and lack of knowledge regarding available mental health services (See Donnelly [19]). Female refugees are frequently identified in humanitarian reporting as being particularly vulnerable to physical and mental health difficulties and having unique health care needs (see UNHCR [16,21]). Hence, the importance of gender for evaluating healthcare provision and allocating resources appropriately.

Moreover, although the mass influx of refugees to Europe in 2015 and 2016 has been described as a “crisis”, there are several important antecedents. For more than a decade, Europe has received boat migrants crossing the Mediterranean. Such flows have informed a small body of relevant scholarship. For example, Padovese et al. (2013) examined the arrival of boat migrants from the Horn of Africa who reached Malta between 2010 and 2011 only to be housed in open centres across the island [22]. However, to date, few have considered their study of the health determinants of some 2216 migrants, housed in 10 open centres on a European Union state in the Mediterranean [23]. Padovese’s study explores the prevalence of infectious disease including tuberculosis and also considers the impact of reception conditions on anti-social and addictive behaviours including use of drugs, alcohol and risky sexual practices. They also record diagnoses of skin diseases (21.9%), respiratory (19.8%), and gastro-enteric diseases (14.2%) among a migrant population which bears many similarities to those crossing today. The authors’ conclusion that the social determinants of migrants’ health in Malta are related to poor living conditions, language and cultural differences, physical and social environment, gender as well as the lack of integration policies and their explicit linkage between non-specific physical symptoms, psychosocial stress and mental disorders, is especially relevant to Europe’s latest refugee inflow (p. 373).

This article seeks to address the above gaps. It examines the effects of migration and reception on health outcomes among asylum-seekers and migrants who reached Italy and mainland Greece between January 2015 and October 2016 during the height of the migration “crisis”. Drawing upon research findings from a UK Economic and Social Research Council (ESRC) funded project [24], it examines the physical and mental health of asylum-seekers in transit and analyses how the closure of borders has engendered health risks for populations in recognised reception centres in Sicily and in Greece. Using mixed methods including a survey and interviews, this study sought to address two principal questions: (1) What is the nature of health provision in formal reception centres in Italy and Greece? (2) What is the impact of the reception system on the well-being of asylum-seekers?

The conceptual framework is informed by Gushulak and MacPherson’s population health-based approach, defined as one that “considers the relationship between migration and health as a progressive, interactive process influenced by temporal and local variables” [25] (p. 1743). The focus then is on the experiences of migrants and asylum-seekers both in transit and in situ in reception centres. Although the idea of reception has been interpreted in European human rights case law which considers that one enters the reception system from point of submitting an asylum application, and one exits the system once a decision has been made, this definition is far from consistent. We therefore take a pragmatic view and suggest that reception begins not from the point that a claim for asylum is made, but from the point of an individual in need of protection being admitted to the protection system.

Another key concept is the notion of vulnerability. While the European Union’s Reception Conditions Directive has come to specify which groups are in need of addition protection, we note that neither asylum seekers nor refugees in general are treated as “vulnerable persons” [26]. Rather only certain sub-categories have been singled out as eligible for special protection. In this context, vulnerable persons are listed as minors, unaccompanied minors, pregnant women, single parents of minors, and victims of torture, rape or other forms of physical, psychological and sexual violence. Subsequently vulnerable status was extended to victims of human trafficking and FGM. Vulnerable groups are similarly defined in the Greek legal framework, which has also specified persons suffering from post-traumatic stress disorder (PTSD), such as shipwreck survivors or relatives of victims, and victims of trafficking. For the purposes of this article, we treat vulnerability in terms of those who may be denied protection and are at great risk from both state and non-state forces.

The first part of this article sets out the research context and describes the geo-political developments which shaped the design of the reception systems of Italy and Greece. We then sketch the function and operations of the reception system in each country before turning to the empirical study. While mixed methods are a common approach to refugee-related research, given the urgent basis for funding this research and the shifting political agendas, we briefly explain the design and the choice of research instruments and their adaptation for the purposes of this study. The results presented are based on a survey of 700 asylum-seekers in Sicily and mainland Greece carried out between March 2016 and November 2016; and further semi-structured interviews with 45 migrants and 50 key informants including civil society groups, activists, and officials from international relief agencies, NGOs, and the governments of Sicily and Greece. This is followed by a discussion of the results and reconsideration of the nature of health services provided to those in the reception system between 2015 and 2016.

## 2. Research Context

### 2.1. Migration Trends: Flows, Blockages and Arrivals

Between 2015 and the end of 2016, Europe received over 1 million non-EU migrants—nationals originally from Africa, Asia and the Middle East who reached Europe by boat. While the majority of arrivals transited through Greece and Central Europe before making their way to host states, above all Germany, by early 2016 large numbers of migrants were blocked en route. The source of the blockage was two-fold: first, the imposition of national border controls in several EU states bordering the countries of first arrival which prevent migrants from continuing westward; and, second, an arrangement between the EU and Turkey sealed off access to Greece from the Aegean and thus reinforced the external border of the European Union. The effect of internal and external bordering drastically reduced numbers entering Greece but it has not stopped large numbers attempting to cross the central Mediterranean. Of the 71,933 sea arrivals from 1 January to 2 June 2017, there were 7369 in Greece compared to 61,165 in Italy.

The decision by Hungary to build a fence along its borders with Serbia and Croatia in June 2015 was followed by further attempts at border management, often through coercive means. Shortly before the fieldwork began, Austria, Germany, France, Sweden, Denmark and non-EU member Norway introduced passport controls. This was followed by the erection of fences along Bulgaria’s border with Turkey, as well as between Slovenia’s border with Croatia, and then in March 2016, along the Greek-Macedonian border by Eidomeni. By the time the field teams were deployed then there was little movement from Greece to the rest of Europe.

The inflow of migrants into Greece was also curtailed. In March 2016, the European Union and Turkey reached an arrangement (described as the “EU–Turkey deal”), which included several elements: (1) a readmission agreement with Turkey; (2) an agreement to relocate a select number of asylum seekers across the EU; and (3) an agreement to resettle a further 22,000 people from outside the EU to the EU Member States. As a result of the border closures and the EU–Turkey deal, tens of thousands of migrants became contained in frontline states, including the more than 15,000 on Greek island “hot-spots” which functioned as processing and detention centres. In May 2016, Human Rights Watch issued a report following its visits to hotspots on Samos, Lesbos, and Chios which recorded that not only had the police failed to stop incidents of violence against women and children but that facilities in all three centres were overcrowded, with significant shortages of basic shelter and filthy, unhygienic conditions [27,28,29,30]. For others who had successfully moved from the islands to mainland Greece, they faced an equally precarious fate. Thousands were left in limbo, unable to move along the Western Balkan corridor from Serbia where the situation was no less bleak.

During the research period (March–October 2016), the UNHCR estimated that there were, at its peak, 62,000 migrants on the Greek mainland, though NGOs estimated there may have been as many as 80,000 [31]. Unlike Greece, however, which was cut off as a result of the closure of the border with Macedonia and as a result of the EU–Turkey deal (implemented March 2016), migrants continued to cross the Central Mediterranean.

**Figure 1:**
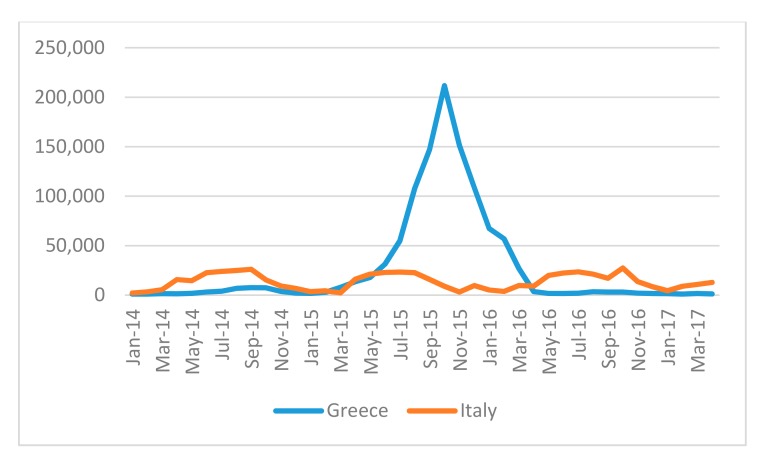
Sea Arrivals in Greece and in Italy. Source: UNHCR/National Authorities in Italy and Greece.

Figure 1 above records migration trends during the research period and notes the spike either side of the EU–Turkey deal.

Migrants seeking to cross the Central Mediterranean are intercepted by naval patrols or private search and rescue missions off the coast of Libya, and usually taken to closed hot-spots (Lampedusa, Pozzallo, and Trapani) or other ports in Sicily and occasionally other locations in Calabria. Once migrants are disembarked in Italy, they can begin the process of seeking asylum, although it should be noted that, during the journey, migrants are effectively profiled by age and nationality. Their health is also assessed while on board the ship.

Although secondary movement is not uncommon, data on numbers intercepted and asylum applications provide a rubric to identify those who may have benefitted from the reception system. In Greece, the number of asylum applications in 2016 was 51,092, three times as high as in 2015 (to 13,195). In Italy, an estimated 123,370 individuals applied for asylum in 2016, while a further 99,920 were pending by the end of the year. The UNHCR records that in 2016 there were 176,000 people in reception centres across Italy as of the end of the year.

### 2.2. The Reception System in Italy and in Greece

In Italy, the formal reception system is multi-layered and includes centres that provide first aid and assistance near sites of disembarkation (CPSA); collective centres set up under a specific Ministerial Decrees for asylum-seekers (CARA); and a second phase where individuals who have already filed an application for asylum are housed in reception centres managed by local authorities (SPRAR). In addition, the Italian government set up Extraordinary Reception Centres (Centri di accoglienza straordinaria) (CAS) to address the shortage of places in first and second reception centres. In practice, however, these extraordinary centres have become the main form of reception. There are an estimated 600 SPRARs hosting 23,000 people while the (CAS) number more than 3000 and host an estimated 137,000 people (February 2017). The reception system in Italy is especially complicated and whereas the Italian Red Cross (Croce Rossa Italiana) screens new arrivals, as migrants enter the reception system, they may come under the operational responsibility of a host of actors. The Italian system relies on sub-contracted service providers working on behalf of national and local authorities.

**Figure 2:**
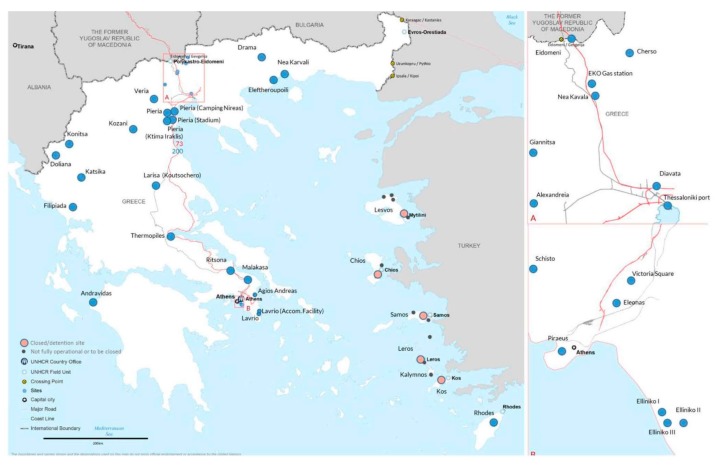
Reception centres in Greece, April 2016.

In Greece, migrants first enter the reception system on the islands, many of which have been designated as hotspots since the EU–Turkey deal. This includes the islands of Chios, Kos, Lesbos, Leros, and Samos. While most migrants are transferred to the mainland (7583 from 1 June 2016 to April 2017), others have found themselves trapped in overcrowded accommodation on the islands which have been the subject of much criticism [32,33]. Refugee centres on the mainland, though formerly under governmental control, are operationally managed by a diverse range of actors including both humanitarian agencies (UNHCR, International Rescue Committee, Danish Refugee Council), and other state institutions. Formally, many centres were directed by the Greek army and national air force. Reception centres are spread across the country and their residents tend to be separated by nationality. Figure 2 shows the spread of reception centres during the peak of the crisis.

The management of refugees and migrants proved to be especially challenging in centres which were organised like traditional refugee camp like structures. The UNHCR grades reception conditions based on the following criteria and provisions.

- Protection including: safe spaces for children; restoring family link services; legal counselling/information provision; mediation to prevent tensions with host community or between communities on site.
- WASH including: the provision of toilets; separate toilet areas for women; showers, including showers with hot water and facilities separated for women; water taps on site; hygiene promoters; regular cleaning of wash facilities; garbage disposal waste management.
- Food provision as ranked by: frequency of meals; types of food provided (hot, sandwiches, dry food); nutritional screening; separate facilities for breastfeeding.
- Health provision as ranked by: distance to nearest health facility; the availability of psychosocial programmes and health referral service.
- Communications including access to internet, charging plugs; two way communications system
- Information—including information regarding: health services; relocation; asylum procedures; food distribution; shelter allocation; restoring family links; UNHCR services and local NGO services.

The UNHCR has also recorded marked differences between the above reception structures. Migrants have experienced a range of housing situations, with many complaining of overcrowding, lack of access to separate facilities for washing, in addition to limited access to lawyers and education. Some of the worst accommodation and ad hoc sites such as Elliniko, the old Athens airport, were closed in 2017. Instead, from summer 2016, asylum-seekers have increasingly been placed in shared accommodation, both state-controlled and private. UNHCR has also financed an urban accommodation scheme on the Greek mainland primarily for those waiting to be relocated and/or classified as vulnerable. Since its inception, 27,742 individuals have benefited from the scheme with 17,081 being housed in April 2017 [See UNHCR (27)].

## 3. Methods

In June 2015, the research team conducted a preliminary visit to Sicily (Catania, Caltanissetta, Lampedusa, Palermo, and Pozzallo) to observe the state of the search and rescue effort and to gain more knowledge about the reception system in Italy. At the time the EVI-MED project was conceived [32], the nature of flows across the Mediterranean was shifting dramatically and there was a marked rise in spontaneous arrivals across the Eastern Mediterranean. The project team therefore decided to include Greece in its proposed study but to limit their investigation to the mainland, not the islands where much research activity was already taking place.

The research team proposed using mixed methods to explore the impact of reception on health service provision and health outcomes. After an inception period in autumn 2015, training workshops were held in Athens, Thessaloniki and Palermo. All researchers were informed of the requirements outlined in the ESRC Framework for Research Ethics which details procedures for respecting anonymity, confidentiality, consent, and handling data. Consent forms were drafted and translated into several languages. Researchers were alerted to the possibility that it might not be possible to obtain written consent, in which case they would seek verbal consent. All accepted that no pressure should be placed on individuals to participate and that participants could withdraw at any point. It was also agreed that any visual material used or produced would ensure that subjects could not be identified without their consent.

During the first stages of the project the researchers relied on UNHCR’s data and other official sources including Eurostat as well as statistics produced by the national statistical offices of Greece and Italy. Data provided by Frontex, the EU’s border management agency, though useful, came with additional complications. One problem was that their data refer to the previous three-month period and record each and every breach/interception/border crossing. The research team was therefore aware that Frontex’s data carried the risk of over counting arrivals and therefore did not rely on this dataset to estimate arrivals when preparing the sample frame. Many NGOs also produced helpful datasets, above all, MSF. UNHCR’s documentation also became more detailed and while there were some small differences between the data produced by the UNHCR and IOM, these narrowed considerably during the project. However, these data sources also included limitations including the lack of gender disaggregated data, especially in the context of minors.

To address these concerns, an original survey was designed and administered as a face to face questionnaire. At the time the project was conceived (May 2015), more migrants were reaching the Central Mediterranean than crossing the Aegean. The research team had promised the funding body that it would conduct 300 surveys in Greece and 400 in Sicily, though this breakdown did not represent the volume of flows as the project progressed and significantly more arrived via Greece. Despite this change, we recognised that the increase in arrivals to Greece was dominated by Syrians and that the team could still gather a useful picture of inflows by limiting the number of surveys to 300 and by oversampling to include non-Syrian populations.

The sample population included third country nationals who had been unable to move on or were currently in the process of seeking asylum, having received subsidiary protection or were without status. To provide a more representative study, a sampling frame was constructed based on the most recent UNHCR’s data on the nationality of migrants who had arrived. The survey was translated into local languages while field locations were identified by local partners—the Greek Council for Refugees in Greece and Borderline Sicily. In Greece, the research took place in Athens (Elliniko, Eleonas, and Piraeus) and Thessaloniki regions (Diavata and Eidomeni). In Sicily, research was conducted in Aidone-Enna, Bagheria, Caltanissetta, Catania, Cava d’Aliga/Scicli, Chiaramonte Gulfi, Palermo, Piazza Armerina-Enna, Ragusa, Siracusa, and Vittoria.

The aim of the survey was to examine the functioning of formal reception centres and other types of accommodation provided to asylum-seekers in Greece and in Italy. Developing the population health based approach, the project also introduced a temporal dimension which recognised the dynamic nature of flows and provided a further basis for comparing trends in migration. While flows were especially changeable before the closure of borders in March 2016, even after this date, the stock of migrants fluctuated considerably. With this aim in mind the survey research was designed to be conducted over two periods during 2016. The rationale for dividing the research into two periods (wave 1: March–May 2016; wave 2: September–October 2017) was further justified on the grounds that it would provide a methodologically more sophisticated approach to understanding the way geopolitical pressures affected both the entry and exit of third country nationals.

They survey included a mix of multiple choice answers and some open/write-in answers. Survey questions sought to gather information on demographic characteristics, migrant well-being, health and disability; routes taken and last country of residence; the use of agents, smuggling and means of arrival; experiences of the reception process and assistance provided; and plans and expectations for the future. With respect to health specifically, the survey posed five questions. Participants were asked to identify themselves as having a serious health condition, if healthcare was a factor influencing their flight; whether they contracted any illnesses/diseases in transit; if they received health support; and if they received psychological support and/or counselling.

A short pilot study was conducted in December 2015 and full fieldwork began in March 2016. Field teams quickly found that certain populations were difficult to capture in the survey. For example, in Italy, where Eritreans were treated as a privileged group, we found that their experience upon arrival contrasted with other nationals that were processed in hot-spots, upon disembarkation. Most Eritreans were immediately transported from Sicily to centres in mainland Italy and therefore were not available to participate in the survey. We therefore asked the local teams if they could use their local knowledge to identify some Eritreans, recognising that this approach was far from random. We also included in the survey a mapping exercise. All participants were asked to mark their journeys on a map and were questioned regarding their route and transit countries.

In addition to the survey research, the research team conducted in-depth interviews with asylum-seekers (45) to gain more contextual knowledge about the functioning of the reception system. The research team oversampled some understudied foreign nationals in order to gather more information about how their experiences of reception and their prospects of asylum, relocation, resettlement, or eventual return compared to groups that had been prioritised for international protection (above all Syrians). In addition, the research team sought where possible to note gender discrepancies and through the in-depth interviews to explore the role of gender on the experience of reception.

Further interviews were conducted with key informants (50) including representatives of government offices, humanitarian and relief agencies, NGOs and activist organisations, in person and by phone, in Brussels, London and Rome. The stakeholder interviews offered the research team the opportunity to explore how reception operates in each country and feeds into the national asylum system and related policies. We were especially keen to learn about the operation of the EU’s relocation and resettlement programmes, and possibilities for family reunification further to Dublin Regulation. Interviews were conducted with agencies including Frontex, the UNHCR and IOM and their partner NGOs and civil society organisations. We also interviewed government agencies responsible for search and rescue, immigration and asylum, as well as relocation officers from participating EU Member States.

Upon completion of the fieldwork, survey data were entered into a shared online system (QUALTRICS) and were cleaned to remove errors and address missing variables. This produced a dataset of 700 individuals which was analysed using SPSS (IBM, Armonk, NY, USA) to identify frequencies and to explore the impact of age, nationality, gender and health, among other variables, on the migrant experience before (i.e., in transit) and during the reception process. Interview data were transcribed and coded to explore key themes, as discussed below.

## 4. Results

The section below presents the results from the survey in response to specific questions. The results are organised to inform the nature and national composition of the survey participants, their reasons for leaving and experiences in transit. Responses to the survey on key points are further illustrated with evidence gathered in interview. The issue of health was most relevant in the context of the reception centre setting, though it did also come up in both interviews and in response to specific survey questions and in interview regarding the quality of healthcare provided inside the reception centres.

### 4.1. General Observations

The survey results provided much information on the composition of groups in both countries. In the case of Italy, migrants were principally from West Africa and Eritrea. In the case of Greece, they included nationals from refugee producing states, above all Syria, Afghanistan, Iran, and Iraq; in addition to Pakistan. Flows to Greece were also less diverse. The top four nationalities in Greece comprise 91% of arrivals in 2016 while in Italy the top seven nationalities comprised 65%.

Table 1 above records participants in Italy and Greece, respectively and their countries of origin.

One defining characteristic of flows is the spread of nationalities, with Afghans, Iraqis, Iranians and Syrians almost exclusively present in Greece and absent from Sicily. Nationals from Nigeria, Gambia, Mali and Eritrea are heavily present in Sicily and are conversely absent from Greece. The project team also noted the increase in Eritreans and Guineans further to increasing levels of instability of those countries while the number of Nigerians and Syrians also rose. Only Pakistanis are present in large numbers in both Greece and Sicily. However, it is important to point out that the presence of Pakistanis was far from standard. The mapping exercise produced information which suggested that some Pakistanis present in Sicily has not arrived by boat crossing the Central Mediterranean but that they had taken the Aegean route and then travelled down through the Balkans to Italy and eventually to Sicily. Their presence therefore challenges prevailing accounts of Mediterranean migration which emphasises flows along one of two routes.

**Figure 3:**
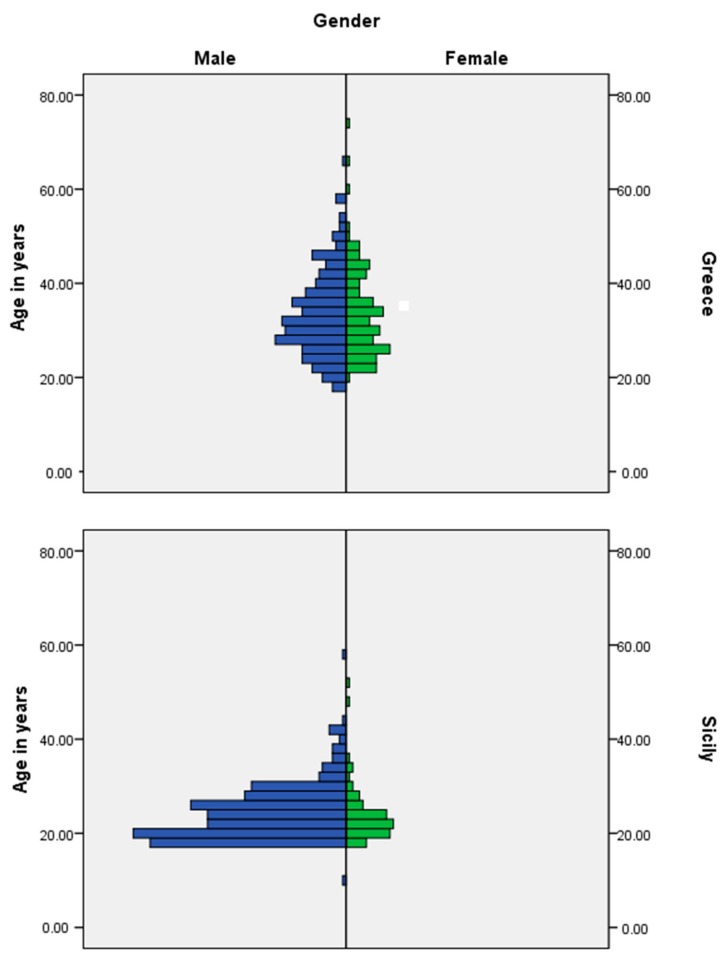
Participants by country of arrival, gender, age and wave.

There were important socio-demographic differences between arrivals in Italy and in Greece as represented in Figure 3.

In Sicily, the presence of predominantly single young men between the ages of 18–30 accounts for the majority of arrivals. In Greece, by contrast, both men and women are present in more equal numbers (64% male to 46% female), and the age range is greater. The results show that it was not just that there are more women in Greece, but that they brought with them their children and were likely to travel with family members. The largest presence of migrants by nationality and gender is in Greece where almost 70% of Afghans were male, in contrast to 30.2% females. Syrians by gender appear almost in equal numbers (51% male; 49% female). In Sicily, while overall the population concerned is largely male, there is a sizeable percentage of Nigerian females (almost 40%). In both waves, only three per cent of participants described themselves as pregnant though in each wave a number of women had given birth recently.

**Figure 4:**
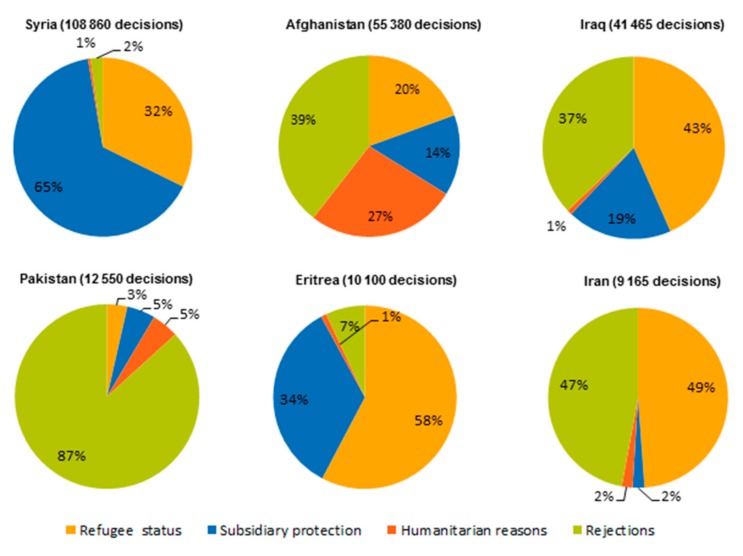
First instance decisions in the EU-28 by outcome, selected citizenships, 4th quarter 2016. Source: Eurostat May 2017.

There was also a marked shift in the recognition rates for asylum-seekers during the research period. In 2015, an estimated 97.2% of Syrians and 89.8% of Eritreans received asylum. As indicated in Figure 4 below, some countries also prioritised particular nationalities for asylum; for example, Italy recognised a greater percentage of Eritrean claims than other Member States.

As recorded in Table 2, recognition rates changed as Member States became increasingly more selective with the introduction of both European policies and tighter restrictions on entry.

### 4.2. Inside the Reception System

The reception systems of the two countries were noticeably different. Living conditions in Italy were relatively better than in in Greece where participants lived in a range of institutions, from insecure camp-like settings to shared accommodation in modern apartment blocks. The research team witnessed some truly shocking living conditions in Elliniko (Athens) where Afghans were housed in the disused airport, football/baseball and hockey stadiums and lived inside tents or makeshift partitioned spaces. While Elliniko was condemned, for some participants, this setting was an improvement. One Afghan participant said she had previously been forced to live in one of 70 tents outside the formal centre because she did not have a “special stamp”. As a result, she and the other tent dwellers were initially denied food and access to the centres in Elliniko. Being moved into the centre, however, brought other challenges. Accounts of violent crime and fears of gender-related violence were also reported. In other parts of mainland Greece, refugees were housed in better conditions; while many had lived in tents, eventually air-conditioning units were installed. During the research period, residents were gradually moved into apartments and shared living spaces with support from NGOs.

In Italy, we found that, in spite of different bases in law, in many cases, there was no difference between certain types of reception centre; for example, the differences between SPRAR and CAS were not always evident. Most of those interviewed had experienced living in multiple types of accommodation until they were settled and could apply for asylum. In Sicily, some participants described how they had been moved from small villages to towns and in some cases cities like Catania where they lived in multiple occupancy rooms which were “good enough”. Many others described how they had been sent to remote villages with very little possibility to do any activity which would advance their integration. For them, there was limited opportunity for language lessons or training. The few unaccompanied minors we observed reported to our NGO partners that they generally fared better and spoke about how they received language instruction in Italian. In addition to activities including sport, they received and phone cards.

### 4.3. Examining Involuntary Flight 

The survey data record that the overwhelming majority of participants were in fact forced migrants fleeing persecution, war, famine, and personal insecurity. This information was further recorded in the interviews where participants often went into great detail to describe their life beforehand and their reasons for fleeing. Most participants gave multiple reasons for leaving and seeking to enter Europe. One Syrian interviewed in Greece put it simply—”I used to live with my wife in Idlib. We had a normal life there until the outbreak of war. Our house was bombed and we lost everything. After that we hadn’t any option but to leave our country” (Syrian man, aged 47). In addition to refugees fleeing Syria, Iraq and Afghanistan, several spoke about how conflicts in Cote d’Ivoire, Mali, Sierra Leone drove them out in search of security. Some participants interviewed in Greece spoke about how they fled Islamic militants including the Taliban in Afghanistan and Daesh/IS in Iraq. Often, insecurity was magnified by other pressures, including inter-ethnic tension and gender-based discrimination and violence. As one Afghan woman from small village reported:

*“In June 2015 my father was pressed by the Taliban to offer me or my sister to them. He asked them for a couple of days in order to be prepared and decide which of us had to go. In the meantime I went with my sister to my uncle’s family in Kabul. When the Taliban returned and did not find us, my father was killed. Immediately after this incident my mother joined us and after a few days we left the country to save our lives. We couldn’t think to stay in Afghanistan because now we are a family without a male, which means that we have no protector. We cannot work, we cannot go out alone, we cannot survive without a male”*.

(Afghan woman in Greece, aged 26)

As indicated in Table 3 below, fear and insecurity drove our participants to Europe in large measure, only 18% described their motivation as economic and an even smaller number said they came for personal or post-materialist reasons like travel or study. This finding affirms other research which claims that most of those who reached Europe during the research period were drawn by “push” rather than “pull” factors and represent a predominantly asylum-seeking population. 

How and why people reached Italy and Greece is a more complicated story and many of those surveyed reported they had no control over their eventual destination. This was especially the case for those who took the Central Mediterranean route and had journeyed via Libya. In particular, we found that several West Africans had hoped to work in Libya which was relatively stable at the time when they began their journey but that then changed. As one Senegalese migrant reported in interview,

*“Well I am here (Sicily) but it was not my option to come here. My initial choice was to go to Libya…I found myself stuck in Libya, I could not go home. I can guarantee you that amongst us, whether it be a refugee or an economic migrant, 95% of us are stick against our own will. Once you arrived in Libya it’s better for you to cross the Mediterranean Sea than to turn your back to save your life. He explained that often people were obliged by forced to leave at the risk of death”*.

(Senegalese male aged 18)

Some participants were simply caught up in the “crisis” as a result of personal accident. For example, one female from Morocco who married a Syrian in Turkey in 2016 arrived in Greece with her husband where she gave birth. While he was entitled to be relocated and travelled on to France, she was left behind in Greece because of her nationality. Those who reached the Greek islands and were eventually moved onto the mainland considered it to be a transit point. Almost 63% of respondents of our respondents recorded that they never intended to remain in Greece but had hoped to move on.

### 4.4. Traumatic Experiences: Torture and Exploitation in Transit

The survey results also record shocking instances of abuse, especially for those who had travelled across the Sahara via Libya before boarding vessels for Sicily. Participants who transited through Sicily (52%) reported that they experienced mistreatment during their journey both overland and at sea (in contrast to 16.4% in Greece). One Guinean recalled how Libyan rebels tore up his documentation and threw him in jail for two weeks. As he admitted, “I had no clue that in Libya people were being imprisoned, brutalised, killed and tortured. For example, people who tried to escape from prison were shot dead right away in the most inhumane ways. They were slaughtered. I witnessed people being beaten up to death”.

Accounts of kidnapping, physical and psychological abuse and imprisonment were not uncommon. A man from Cote d’Ivoire who was interviewed in Italy reported that he had spent one year and nine months in a Libyan prison. One West African migrant even recounted how he was stabbed by a policeman in Libya. Explaining to the team interviewers how he received a noticeable scar, he provided the following account:

*“I had in while I was in prison; it was a policeman that stabbed me there. He asked me…that was a Thursday morning, on a Thursday morning, there was a 6 square metre room, we were 138 in this room, 138 in a 6 square metre room, 6 square metre I am telling you, we were 138 confined in this room…what we did… there were hours where you would need to go toilets especially around midday, if you needed to go toilet, you would need to wait up to 7 PM or even the next morning”*.

(Senegalese man aged 18)

Others were detained upon arrival in Libya. This was most pronounced among migrants from the Horn of Africa who reported arrest or detention in transit. While 31.7% of participants in Sicily had been engaged in paid work as they made the journey across Africa, some 17% of others explained that they were coerced to pay smugglers and to finance their onward travel. The same applied to participants who travelled via Turkey, though in smaller numbers. As one Moroccan graduate in English literature who had left her country in search of a better life, explained:

*“We arrived in Turkey in January 2016. In Turkey I had the chance to work for about 2 months as a street seller. The amount of 6000 euros my father had to pay the smugglers, was not enough for me to continue the trip to Europe. So, I had no way but work for a while and find the way to leave”*.

(Moroccan woman, aged 26)

Table 4, drawn from the survey results, records that many more migrants principally from Africa worked in-kind, often under exploitative conditions, to pay for their journey across the Mediterranean. The prevalence of smuggling and in some reported cases trafficking along the Central Mediterranean route in Libya and elsewhere in Sub-Saharan Africa is further evidenced in the interviews.

### 4.5. Health Issues as a Motivating Factor and in Transit

A small number of participants listed healthcare as one reason for moving on, though this was not a major factor in their decision to reach Europe. More importantly, participants recorded that their health was directly affected by their journey. Almost 40% of participants recorded that they contracted diseases and illness in transit. This was most evident for those who took the Central Mediterranean route who reported a number of ailments. As illustrated in Table 5 below, the most prominent illness was scabies followed by dehydration, diarrhoea, physical injuries, tuberculosis, and pneumonia.

Many participants wrote in additional medical complaints which tended to be associated with the journey as well as living in camp-like settings. Illnesses reported by our respondents included respiratory issues, cold and flu, dermatological issues and either minor illnesses, often due to the difficult conditions of travel, but also some severe health issues such as leukaemia, hepatitis or Alzheimer’s disease, as well as various degrees of psychological distress.

### 4.6. Torture, Abuse and Mental Health 

The interviews recorded several cases of psychological distress due to torture or severe mistreatment. Some reported that they suffered from mental health problems at various stages of their journey; though more precise information was provided in interview which clarified which events and circumstances most affected their well-being. Many of those who had experienced torture or the direct effects of war and violence, recorded that their mental health was negatively affected. Equally, several identified detention on the Greek islands as negatively affecting them. Such factors were not however limited to those on the islands but were even noted in more open centres on the Greek mainland. As one MSF psychologist explained:

*“Prolonged stress causes some symptoms, usually psychosomatic. People come with this kind of problems… I have a headache, I can’t sleep, I don’t have an appetite et cetera. It is a problem of adaption. It is obvious why. Who could adapt to these conditions I mean? And stress about what is going to happen, about my children, are they going to get in? That is why they left Iran. They were second class citizens in Iran, their children couldn’t go to school, they couldn’t get decent jobs so they left to find a better future. And now they are in a halt and what is going to happen. So not being able to sleep due to ruminative thinking about what is going to happen et cetera. This is very common. And then there were people who already had a predisposition to not being mentally healthy which the situation worsened”*.

(MSF Ellinko 7 July 2016)

In Italy, we also identified profound mental health challenges among asylum-seekers, though most were not linked directly to the process of reception. Rather, we suggest psychological trauma was inflicted in transit. This finding was supported by a parallel study conducted by MSF during the research period. MSF screened 385 individuals in Ragusa province and found that 193 (50%) presented mental health problems above all, PTSD (31%), and depression (20%). They suggested that, among the many sources of trauma, patients who had been in combat situations at risk of death or had witnessed violence or death and had been in detention were most likely to present symptoms of trauma [33]. These findings are indicative of the pervasive human rights abuses that confront migrants, especially as they seek to transit via Libya.

### 4.7. Access to Healthcare and Health Risks within the Centres

In Italy, in many cases, refugees and migrants were housed in institutions where medical staff were not routinely present. Participants did however have access to doctors in the local town and would be escorted by a SPRAR staff member in order to attend appointments. Their situation was considerably better than what was on offer in Greece where, despite a new law which opened up access to the public health system to vulnerable people including beneficiaries of international protection, asylum seekers and their families (Law 4368/2016 of 20 February 2016) [34], we found much irregularity.

Just over half of participants in Greece received access to health services (57% in contrast to 43% who had no access). Once inside the reception centres participants were forced to rely on an overstretched system [32]. The way in which medical complaints were addressed depended largely on geography and the resources available within the centres. Though most reception centres in Greece had a medical referral system on call seven days a week, UNHCR recorded significant disparities between the types of services afforded to refugees, again in part due to the location of centres.

In Greece, the government appointed centre manager who was responsible for keeping registries of residents had considerable influence over the allocation of medical and health resources. The manager was often a representative of another organisation, such as the UNHCR, and while they coordinated with the government on a day to day basis, NGOs often initiated additional medical interventions. Medical provision was therefore uneven and access uncertain. The issue was more pronounced in large facilities where it was not simply access to medical assistance and supplies but basic hygiene was challenged by overcrowding. As one MSF worker based in Elliniko explained, one major challenge was keeping new-born babies clean and preventing infectious and diarrheal disease. However, she added, that many of the predominantly Afghan mothers were highly experienced and they “somehow managed” despite the deplorable conditions (MSF Elliniko, 7 July 2016).

### 4.8. Access to Psychosocial Services

Psychosocial support was especially limited. In Greece just over one quarter of participants surveyed (26%) reported that they had access to psychological services. In Italy, there were also significant discrepancies between the type of services provided. While 74% enjoyed access to health services in Italy, only 35% has access to psychological services. These shortcomings were reflected in responses to the survey with most participants unaware of health and psychological services nominally provided in centres.

“(Participant)—Yes, there is a psychologist here in this centre but I was never introduced to him

(Interviewer) Could you remind me again since you arrived? You said 9 months right? And you still have not been introduced to him?

*(Participant)—It is now 1 year and 4 months since I arrived in this refugee centre…but if there is a psychologist here, personally, I do not know him, they never, never introduced me to a psychologist here. The only persons that I know here are these two persons Francesca Pina and the manageress, only them, only these two persons that I know here”*.

(Sengalese man, Italy 2016)

Another Gambian man similarly responded that he did not know if there was a doctor on site—”No I don’t know, they told me about the doctor but I don’t know”, (Gambia, Male, 20 Italy). Such uncertainty was reflected across the research sample, as recorded in Table 6 and Table 7 below.

It should be noted that such variation in service provision between reception centres was evident from the point when the reception system was first introduced, as indicated in Table 8.

### 4.9. Gender, Medical and Psychosocial Provision

The relationship between gender and health provision was raised in questions posed to recent mothers and to women who identified as pregnant. In response to the questions “*would you describe yourself as being pregnant?*” and “*do you receive health support?*”, all but one participant responded positively. With respect to counselling, however, the participants who were pregnant responded overwhelmingly that they did not have access to psychosocial assistance. In fact, only one out of 14 pregnant respondents recorded that she was receiving counselling and psychological support. Counselling as a service was generally absent and only those who attended clinics outside the camp appeared to have any access to psychosocial support.

### 4.10. NGOs Filling the Gap

Key organisations such as Médecins Sans Frontières (MSF), the Hellenic Red Cross, Women and health Alliance (WAHA), and Médecins du Monde worked to fill the gap. These organisations could be found in reception centres both on the Greek islands and mainland, often working in parallel with other relief providers such as the Danish Refugee Council. MSF in particular had a considerable presence in the centres having 700 staff dedicated to Greece on top of its 27-person national office. For MSF, Greece was its second largest mission after Congo (MSF Interview 4 July 2016). In 2016, MSF medical teams in Greece carried out 72,740 health consultations, including: 8207 mental health consultations, 3195 sexual and reproductive health consultations, 61,338 medical consultations, covering primary healthcare, physiotherapy, treatment for chronic diseases and more [33]. We found in the Ellinko “Arrivals” on the site of the former airport, MSF had set up two mobile units: one van providing midwife led sexual and reproductive health services, and the other van consisted of three psychologists who provided mental health and psychological services. Following a health promotion campaign, MSF could vaccinate children as young as three weeks up to 15 years of age in the three camps over the course of three days.

However, despite their commitment to Greece, the presence of medical provision by NGOs is far from universal and access to healthcare varies significantly.

## 5. Discussion

The above study was conducted during a period when the agenda of the European Union was changing rapidly. The closure of borders among EU member states and the implementation of the EU–Turkey deal created many operational challenges including the relocation of migrants from the Greek islands to centres on the mainland. While the population appeared to stabilise, in practice we noted that during the research there was increased pressure to move people out of centres and into shared urban dwellings. Similarly, in Sicily, migrants were dispersed across the island. Since asylum-seekers and migrants of the same nationality tended to be housed together, it is possible that, in addition to Eritreans, some nationals of small groups were not identified for inclusion in the research project.

The administration of the survey in waves had many advantages, however we note that the pressure of time and limitations of funding meant that we could only divide the project into two waves. The fact that migrants have continued to cross the Central Mediterranean and that new flows are arriving from Morocco and Egypt affirms the highly dynamic nature of migration and the relevance of comparative research of this nature.

Health was one of several themes explored in the survey and was limited to just five key questions. Although further interviews were conducted to gather more information on this theme, the number was limited on account of time. Further in depth interviews would strengthen the evidence base and also draw out more information about the long term impact of being housed in reception centres.

Despite the above limitations, the above findings present an important record of the functioning of the receptions systems and the provision of health services to asylum-seekers and migrants. In the first place, we note marked variation between the flows of the two main receiving states, Greece and Italy. We identify two distinct sub-systems characterised by significant differences in terms of the national and demographic composition of migrant groups, gender, age, and many social characteristics. The differentiation occurred during a period of marked geopolitical developments which aimed to curb both inflows and outflows of migrants and refugees.

We record that approaches to reception differ across the two states and that these differences may be partially explained by the countries’ experience of asylum management. For example, in contrast to Greece, Italy which has long been a receiving and destination state has had legislation in place for over a decade that provides for the creation of the reception institutions where asylum seekers are housed. However, in practice, there is no uniform reception system in Italy and migrants may be placed in a number of institutions. More telling is the impact shift in gender. In Greece, over summer 2015, the percentage of women and accompanying children began to increase which is reflected in the difference between the two sets of participants (“stock”), where the presence of men in Sicily greatly outnumbers women, the situation in Greece is remarkably dissimilar and reflects a pattern of family migration recorded elsewhere in the surveys.

Although healthcare was one minor reason for moving on, health issues feature at all stages of the journey and throughout migrants’ experience of the reception system. This finding affirms the relevance of Gushulak and MacPherson’s population health-based approach. In this setting, local variables include the widespread abuse of human rights in countries of origin such as Syria, but also in Libya and in Greece where policies of detention have caused significant distress. In terms of physical ailments, participants who had transited via Libya recorded multiple illnesses which is in part informed by the longer and more dangerous journey across the desert. However, in both Italy and Greece, participants recorded that their health was directly affected by their journey. The above findings in Italy also align closely with the earlier study of health determinants in Maltese reception system by Padovese et al. (see [23]), which found several of the same ailments recorded along the Central Mediterranean route. While the physical diagnoses were dominated by skin diseases such as scabies, the above account clearly provides evidence that the journey to Europe had a negative impact on participants’ health and affirm previous findings note the effects of forced migration and the inter-related sources of morbidity, stress and trauma.

The most enduring problems recorded were psychological and mental health issues, with high incidences of depression. The causes include traumatic experiences pre-flight for example torture both in country of origin and in transit. Further, the devastating effects of losing family and being separated from family and local surroundings exacerbated their trauma. Such conditions appeared to worsen with longer periods spent in the reception process.

The effects of gender on health and access to services requires further consideration, as does the concept of vulnerability. Although female participants presented symptoms of stress and depression associated with their flight from Syria, so did male participants. In Italy, where there was a much larger presence of single males, we also note the effects of torture and bonded labour experienced in Libya. The lack of psychological services was largely universal, though within the centres, women were more vulnerable as a result of gender-related violence, sexual assault and general insecurity and thus may most benefit from counselling and psychological services. In addition, we note that maternal health was compromised by the sub-standard centres in Greece. Afghan mothers who were housed in the worst centres were particularly disadvantaged by the availability of health care. In this setting, we note the limitations of the category of “vulnerability” used by the European Union institutions. In theory, vulnerability criteria establish which individuals may be prioritised for assistance; in practice, access to healthcare is uneven and determined by contextual factors.

The wide variation in terms of health and psychological services offered to those inside the reception system is recorded in both the UNHCR’s reports and in the quantitative studies which show that in both Greece and in Italy, there was a significant lack of access to psychological support and uneven access to medical services. Even though doctors were present in the centres, respondents reported having limited access. The fact that participants may have been unaware of medical services provided by NGOs and contracted agencies working within the reception systems points not only to broader issues of resource management but also the need to raise awareness. One possible point of reform lies in the role of the centre administrator who plays a pivotal role in the allocation of resources including initiating and engaging medical interventions. Moreover, we note that, in Greece, there was a tendency for centres to be run in a top-down manner and those housed in centres therefore relied on a chain of relief workers to provide critical information.

The interviews included above further record that participants were unaware of available services, were often unable to access necessary services, and were simply provided with essential services inside the reception centres. Although the level of service differs markedly between Italy and Greece—Italy providing more services overall—the dearth of psychological and mental health services indicates a major lacunae in the management of reception systems in both states.

## 6. Conclusions

Although health features prominently in refugee studies, in the context of Europe’s “refugee crisis”, there has been little attention given to the ways in which health issues have been managed or in the case of psychological services neglected in the context of refugee reception. Given the massive investment by the donor community—the European Commission having allocated €371.16 million to the Greek authorities and international organisations operating in Greece—the uneven provision of medical assistance and especially the dearth of psychosocial support is most alarming [34]. This is particularly notable given that during the research project the number of asylum-seekers in Greece was estimated to be just 50,000. By contrast, flows of migrants to Italy increased substantially. As the above discussion records, the use of overcrowded centres in both countries and especially closed sites like the hot-spots on the Greek islands may increase vulnerabilities and exacerbate mental and psychological stress, thus indicating an important area for reform.

Demographic differences clearly impact on the health needs of refugees in the two countries. Further, the range and quality of provision in Italy is noticeably better in Greece which suggests that, past experience, including the introduction of specific legislation and the development of an asylum infrastructure, may be responsible for this comparative improvement. In the future, Greece too may benefit from its experience as a refugee hosting state, provided that institutional memory is protected and lessons learned.

The fact that medical provision varies from one centre in Greece to another further begs the question, to what extent is this variation due to the quality of centre management and calls for further investigation of the role played by centre managers. There is clearly a greater need to ensure that information cascades down more effectively. The criteria used by the UNHCR to grade health services in reception centres does not address the substantive issue that, even where services are provided, asylum-seekers are not accessing services available. This suggests there is a great need for more information campaigns and further induction sessions to direct refugees to essential services.

By drawing upon research conducted both inside reception centres and with key stakeholders and across two systems in Italy and in Greece where the largest numbers of refugee arrivals have been contained, this article demonstrates that health provision is not as recorded in official UNHCR reporting. Rather, there are critical gaps between provision, awareness, and access to health and psychological services. Above all, we note that the process of reception in Italy and Greece, just as the protracted detention of asylum-seekers in other host states, does not alleviate mental health problems but rather is a contributing factor in the aggravation of illness and trauma among refugee and asylum-seeking populations.

In light of the above, we offer the following recommendations.

It is essential to make health provision more consistent across the two systems in Italy and Greece. In this context, the first priority must be to ensure a more regular and visible presence of medical staff in all types of reception centres.

There is an urgent need to mainstream psychosocial services across the reception systems of both countries.

Recognising that issues of language and interpretation make it difficult for migrants to interact with doctors and other medical staff, more attention should be placed on language training and cultural integration to enable the delivery of effective medical support.

Information is essential to the effective delivery of services. We therefore recommend that the UNHCR and its partners introduce more information campaigns inside reception centres with a view to directing asylum seekers to the health services they require.

The UNHCR and its partners should gather more analytical data, disaggregated by age and gender, in order to inform the delivery of services and allocation of critical resources.

## Figures and Tables

**Table 1:** Participants by survey country, wave and country of birth.

Country of Birth	Greece			Italy		
*Wave*	1	2	Total	1	2	Total
Syria	30.6%	34.6%	32.6%	0.5%	1.5%	1.0%
Nigeria	1.9%	1.3%	1.6%	21.8%	24.5%	23.1%
Gambia	…	…	…	17.8%	13.0%	15.4%
Pakistan	11.3%	11.1%	11.2%	10.4%	3.0%	6.7%
Afghanistan	13.1%	14.4%	13.7%	2.5%		1.2%
Iran	14.4%	11.8%	13.1%	…	…	…
Somalia		0.7%	0.3%	0.5%	2.5%	1.5%
Mali	…	…	…	6.9%	7.5%	7.2%
Eritrea	1.3%		.6%	1.5%	11.0%	6.2%
Iraq	8.8%	7.8%	8.3%	…	…	…
Other	18.8%	18.3%	18.5%	38.1%	37.0%	37.6%
Total	100.0%	100.0%	100.0%	100.0%	100.0%	100.0%

**Table 2:** Legal status (responses by country across the two waves).

	Wave				Wave		
Greece	1	2	Total	Sicily	1	2	Total
Asylum seeker (i.e., already applied for asylum)	19.9%	32.0%	25.9%	Asylum seeker (i.e., already applied for asylum)	64.2%	31.5%	47.9%
Refugee (status recognised)	0.7%	0.7%	0.7%	Refugee (status recognised)	8.0%	1.0%	4.5%
Humanitarian permission		0.7%	0.3%	Humanitarian permission	10.9%	11.0%	11.0%
Subsidiary protection		1.3%	0.7%	Subsidiary protection	1.0%	2.0%	1.5%
Refused asylum seeker	1.3%	0.7%	1.0%	Refused asylum seeker	9.0%	2.5%	5.7%
Irregular	18.5%	12.0%	15.3%	Irregular	2.0%	0.5%	1.2%
Temporary suspension of deportation	49.7%		24.9%	Temporary suspension of deportation			
Asylum seeker—currently applying against a commissions denial		8.7%	4.3%	Asylum seeker—currently applying against a commissions denial		23.5%	11.7%
Asylum seeker—Dublin case		36.7%	18.3%	Asylum seeker—Dublin case		0.5%	0.2%
Other (please specify)	9.9%	7.3%	8.6%	Other (please specify)	0.5%	13.5%	7.0%
I do not know				I do not know	4.5%	14.0%	9.2%

**Table 3:** Why did you leave your last country of residence? (% within country) (respondents could select up to three options).

Reason for Leaving	Greece	Sicily
War	48.7%	23.6%
Persecution or targeted violence	37.4%	48.9%
Concerns regarding family security	45.4%	42.9%
Environmental disaster/famine	0.7%	5.3%
Health care needs	3.0%	5.3%
Work related	10.3%	10.3%
Economic reasons	16.6%	18.3%
Education	2.0%	2.8%
Family reunification	7.6%	1.5%
Exploring Europe	1.0%	1.5%
Other	5.0%	2.8%

**Table 4:** Did you pay for your journey?

Pay for Journey (Yes/No)	Greece	Sicily
Yes—Money	94.0%	65.5%
Yes—In Kind (e.g., through work)	1.0%	16.8%
No	5.0%	17.8%
Total	100.0%	100.0%

**Table 5:** During your journey did you contract any of the following health issues...? (per cent within country).

Illness Contracted	Greece	Sicily	Total
Tuberculosis	0	13	13
	0.0%	3.3%	
Scabies	1	70	71
	0.3%	17.8%	
Pneumonia	5	9	14
	1.7%	2.3%	
Dehydration	18	44	62
	6.0%	11.2%	
Physical injuries	20	50	70
	6.7%	12.7%	
Diarrhoea	3	26	29
	1.0%	6.6%	
None	233	192	455
	77.7%	48.7%	
Other	35	11	46

**Table 6:** Do you receive psychological support and/or counselling?

Country		Yes	No	Total
	Greece	79	220	299
	Sicily	132	241	373
Total		211	461	672

**Table 7:** Do you receive health support?

Country		Yes	No	Total
	Greece	168	128	296
	Sicily	276	99	375
Total		444	227	671

**Table 8:** UNHCR Health Services in Reception Centres 2016.

Site	Distance to the Nearest Health Facility	MoH Psychosocial Programmes Available	Other Psychosocial Programmes Available	24 × 7 Referral Service in Place
Elliniko I (Hockey Stadium)	No	No	Yes	Yes
Elliniko II (West/Olympic Arrivals)	No	No	No	Yes
Elliniko III (Baseball Stadium)	No	No	No	Yes
Eleonas	Yes	No	Yes	Yes
Schisto	Yes	No	No	Yes
Ritsona	No	No	Unknown	Unknown
Larisa-Koutsochero	Yes	No	No	Yes
Fthiotida (Thermopiles)	Yes	No	No	Yes
Doliana	Yes	No	No	Yes
Diavata	Yes	No	Yes	Yes
Nea Kavala	No	No	Yes	Yes
Cherso	No	No	Yes	Yes
Kozani (Leikovrisi Stadium)	Yes	No	Yes	Yes
Filipiada	Yes	No	No	Yes
Katsika Ioanninon	Yes	No	Yes	Yes
Giannitsa	No	No	No	Yes
Veria (Armatolou Kokkinou)	Yes	No	No	Unknown
Konitsa	Yes	No	Yes	Yes
Nea Karvali	Yes	No	No	Yes
Eleftheroupoli	No	No	No	No
Drama	No	No	No	No
Andravidas	Yes	No	No	Yes
Eidomeni	Yes	No	Yes	Yes
Victoria square	Yes	No	Yes	No

Source: UNHCR 2016.
